# Gender Differences in the Associations Between Informal Caregiving and Wellbeing in Low- and Middle-Income Countries

**DOI:** 10.1089/jwh.2019.7769

**Published:** 2020-10-14

**Authors:** Nandita Bhan, Namratha Rao, Anita Raj

**Affiliations:** ^1^ Center on Gender Equity and Health, University of California, San Diego, La Jolla, California, USA.; ^2^ Center on Gender Equity and Health, Department of Medicine, University of California, San Diego, La Jolla, California, USA.

**Keywords:** informal caregiving, mental health, gender equity, caregiver burden

## Abstract

***Background:*** Health risks among informal caregivers have received inadequate attention in low and middle income countries. We examined cross-sectional data from 28611 adults 18 years and older in Ghana, India, Mexico, Russia and South Africa in the WHO Study on Global AGEing and Adult Health (SAGE) to examine gender differences in informal caregiving and wellbeing.

***Methods:*** Wellbeing was measured by self-rated health, difficulties with tasks, self-reported and diagnosed depression and anxiety. Informal caregiving was specific to adults and constructed as categorical variable with the respondent as: the main caregiver, non-caregiver but an adult in the household needs care, and no-one ill in the household; multinomial gender-stratified regression models assessed adjusted relative risk ratios (ARRRs).

***Results:*** Female caregivers were more likely to report moderate difficulties with life tasks [ARRR = 1.45 (95% CI: 1.01, 2.08)], feel mild-moderate anxiety [ARRR = 1.64 (95% CI: 1.22, 2.22)], and report feeling severely depressed [ARRR = 1.86 (95% CI: 1.28, 2.69)] compared to female non-caregivers. Even when women were not caregivers, having someone ill at home was associated with extreme difficulties with life tasks [ARRR = 2.32 (95% CI: 1.33, 4.04)]. Male caregivers, compared to no-one ill in the household, were more likely to report mild-moderate anxiety [ARRR = 1.8 (95% CI: 1.2, 3.7)] and severe-extreme anxiety [ARRR = 2.22 (95% CI: 1.07, 4.6)].

***Conclusions:*** Caregiving for older adults results in greater health burdens, particularly mental health, for both women and men, though evidence shows that these burdens may be prominent and manifest in more diverse ways for women relative to men.

## Introduction

While the transformational role of community health workers to health systems has received global recognition, the role played by informal (family) caregivers toward the support and care of the elderly and/or to adult family members with long-term health ailments goes largely neglected and unacknowledged.^1,2^ In low- and middle-income countries (LMICs) particularly, increasing populations of older persons, inadequate health systems infrastructure, and emergence of noncommunicable diseases have led to a majority role in the care for the ailing and the elderly (including personal care, financial support, and nursing) being provided by the family members.^3^ In countries like India, research studies estimating the morbidity burdens from the strain of caring for diseases like stroke ^4^ or Parkinson's disease^5^ have been increasing. In other contexts (*e.g.*, Ghana, Russia), economic burden of caregiving and the benefits of home-based versus institutional care are emerging social policy issues.^6^ Caregiving among migrant households in many countries is associated with a clash of cultures or values around balancing different caregiving beliefs.^7^

In both high-income countries (HICs) and LMICs, data are showing that the informal caregiver workforce predominantly comprises of women.^8^ Between 57% and 81% of informal caregivers (for the elderly) are women as per some estimates,^8^ and these usually include wives, daughters, or daughters in law depending on the social context and family arrangements.^9,10^ Economic value of informal caregiving has been estimated to a staggering $470 billion in the United States in 2013, translating to ∼37 billion hours of care^11^; a large proportion of this may be attributed to women's labor contributions as the majority care provider and is being linked to debates around unpaid care work.

Our present knowledge on the issues, challenges, and interventions for informal caregiving draws from the research on aging, noncommunicable diseases, and mental health,^12–17^ where informal caregivers are “*essential partners in the delivery of complex health care services.*”^13^ There is some recognition that conflicts between personal goals and care obligations for family members over long periods can lead to caregiver stress. In this study, research needs to make a distinction between stressors experienced in caring for the elderly versus caring for those ailing for long periods (*e.g.*, dementia or cancer). While care needs have increased in LMICs, few formal institutions and services exist and responsibilities of unpaid care falling disproportionately on women and are considered part of their domestic labor as per traditional norms obstruct their labor force participation.^18,19^

Long periods of caregiving are not only associated with financial costs (including lost opportunity costs), but with health and wellbeing impacts in HICs.^13,20–23^ Effects of caregiving on health can be conceptualized using Pearlin's *stress process* model.^3^ Pearlin conceptualizes stress (as a threatening or burdensome exigency) leading to outcomes and mediated by caregivers' access to resources or opportunities. A diversity of emotions are experienced in caregiving, such as exhaustion, guilt, anxiety, fear of loss, and isolation, and caregivers can feel unsupported and unprepared, experience loss capacity in being able to prioritize and achieve personal goals and ambitions.^3,14,24^ Social institutions, status, and gender roles can influence stress exposure and be associated with outcomes. Case reports and intervention studies have shown gender patterning in care responsibilities, experience of stress, and coping resources.^8^ Coping resources and stress experienced may also be determined by socioeconomic status at individual (*e.g.*, education, incomes) or contextual levels (*e.g.*, residence in rural versus urban setting) and in accessing social network opportunities (*e.g.*, social connectedness). These questions, to date, remain unexamined in present studies.

In this study, we examined gender differences in the association between informal caregiving and wellbeing (or lack of wellbeing namely distress) through the lens of the stress process model using the World Health Organization (WHO)-Study on Global AGEing and Adult Health (SAGE) data from five LMICs. Our main research question examines using gender-stratified models for the associations between informal caregiving and five measures of wellbeing, and the influence of resources and social connectedness. The WHO-SAGE study was conducted in LMICs to understand issues around aging and health^25,26^ and findings from this investigation can add to the growing body of work on gender and unpaid care.

## Methods

### Data and sample

We used data from Wave 1 of the WHO-SAGE (2007–2010).^25,26^ SAGE is a nationally representative population study on health and wellbeing in six LMICs, China, Ghana, India, Mexico, Russia, and South Africa. WHO-SAGE survey is one of the few datasets globally that examines issues related to health and social transitions among older populations and provides crossnational insights into health issues associated with aging. Data from the WHO-SAGE surveys provide a comparative picture across six LMICs. The study was conducted by investigators at the WHO and within SAGE countries. Information on caregiving was not available from China. Hence, the final sample for the study comprised 28,611 participants (Ghana = 5,498, India = 12,118, Mexico = 2,575, Russia = 4,339, and South Africa = 4,081).

### Study procedures for data collection

SAGE was implemented in all participating countries using multistage cluster sampling strategy, with some differences in Mexico.^25^ Study participants were identified from belonging to either of the two categories: (1) persons 50 years of age and older identified from 50+ households; and (2) one person between 18 and 49 years of age identified from an “18–49” household’. SAGE used region and residence to stratify the primary sampling units. In Mexico, the data collection approach was similar, but also included supplementary and replacement samples to account for losses to follow-up (details published elsewhere^26,27^). Study data were collected through face-to-face interviews using standardized instruments, interviewer training and protocols, on health behaviors, chronic conditions, wellbeing and quality of life, and a range of their individual and household socioeconomic determinants. Response rates have varied from 53% to 93% across countries (Mexico: 53%; India: 69%; South Africa: 75%; Russia: 83%; and Ghana: 98%).^25,26^ Response rates were lowest in Mexico attributed to the short time available for fieldwork and inability to conduct revisits.^25^ We accessed open access, publically available data for use from the study website.^26^ The SAGE study has received ethics approval for sharing from the WHO and partner organizations in each country.

### Measures

#### Dependent variables

We used five measures of psychosocial wellbeing representing affect, recognition of own state of health and difficulties, and diagnosed depression. Measures included feeling depressed, feeling anxiety, self-rated health, self-rated experience of difficulties, and diagnosed depression. These measures represented a multidimensional spectrum of health, wellbeing and distress, combining affect/mental health (depression and anxiety), diagnosed depression (influenced by health systems), self-rated health (perception of own health), and difficulties with life tasks (physical challenges faced by the caregiver in providing care). These measures represented distress or lack of wellbeing which were outcomes in the stress process model.

In the study, respondents were asked questions about feeling depressed and feeling anxiety. Feeling depressed was assessed by the question, “*Overall in the last 30 days, how much of a problem did you have with feeling sad, low, or depressed?*” while feeling anxiety was assessed by the question, “*In the last 30 days, how much of a problem did you have with worry or anxiety?*” To ensure full comprehension of the question, data collectors explained the terms “problem” (clarified to explain severity), “sad, low, depressed,” and “worry or anxiety” for greater clarity among respondents. Responses to both questions were rated on a five-point scale of none, mild, moderate, severe, and extreme/cannot do. We categorized these responses as “none” (reference), “moderate,” and “severe/extreme.”

Self-rated health was assessed by the question: “*In general, how would you rate your health today?*” In the assessment of this question, respondents were asked to consider her/his physical, mental, and emotional health, and provide the best estimate for their health. Responses were coded on a five-point scale, categorized as “good” (reference), “moderate,” and “bad or poor” for analysis.

Difficulties with life tasks was assessed by the question: “*overall in the last 30 days, how much difficulty did you have with work or household activities?*” Respondents were asked to consider “activities” as household, work, or school activities, and “difficulty” as meaning having trouble with how usually they were performed. While responding, participants were asked to describe their condition, taking into account both good and bad days in their capacity to carry out an action, irrespective of whether they actually engaged in it or not. Responses were rated on a five-point scale, categorized as “none” (reference), “mild,” “moderate,” and “severe or extreme” for analysis.

Diagnosed depression was assessed by the question, “*have you ever been diagnosed with depression*?” Responses were coded as No (reference) versus Yes. Diagnosed depression may be influenced by health care access (particularly mental health services), socioeconomic status, and social norms. To ascertain validity and sensitivity, we conducted sensitivity analyses and compared diagnosed depression with measures of affect.

#### Independent variable

In the SAGE study, the caregiving module was implemented to understand how households cope and support each other through prolonged illnesses or death. Questions pertained to adult family members needing care due to illness or other reasons. Informal care was described as daily personal care such as help with eating, dressing, bathing, moving around in the house, and assistance with affairs outside the house like transportation to see doctors, going to buy medicine, or managing the ill person's financial situation, health care, emotional wellbeing, or other personal affairs. Respondents were asked if any (one or more) adult family members in the household had needed such care or support in the past 12 months and what support they needed. While the SAGE surveys have been primed to assess issues related to the elderly, this part of the survey examined caregiving issues for those who were seriously ill and required daily personal care (*e.g.*, help with eating, dressing, bathing) as well as assistance with affairs outside the house (*e.g.*, transport to see the doctor, managing financial affairs of the ill person). In each household, information for up to four members who needed support was ascertained and for each person, respondent was asked: “*Who is or was the main person providing care for this adult? Is it you yourself, someone else in this household, or someone outside the household*.” Using this information, we categorized respondents into three groups, if respondent was the *main caregiver* for any or all of the four persons requiring care, *supportive caregiver* for any of these members, or was *not involved in caregiving* because there was on one ill in the household. We created a measure for informal caregiving categorized as “no one in the household was ill and needed care” (reference), “someone was ill but respondent was not the primary caregiver,” and “respondent was the caregiver.”

#### Covariates

We conducted gender-stratified analyses. To adjust for bias, we adjusted for a range of covariates, including age, education, income quintiles, work status, social connectedness, current marital status, and area of residence (rural/urban). Covariates in the study included those that were associated with the outcomes in the study (*e.g.*, age, residence, marital status) and others that potentially mediated the effects of caregiving (*e.g.*, social connectedness). Age was adjusted for in the analyses and the age group 18–49 years was considered as reference compared with participants in the age group 50 years and above.

Current marital status was reported as never married, currently married, cohabiting, separated or divorced, and widowed. We categorized responses as never married (reference), currently married/cohabiting, and separated/divorced/widowed.

Respondent work status (ever) was assessed by the question: “*As you know, some people take jobs for which they are paid in cash or kind. Other people sell things, have a small business, or work on the family farm or family business. Have you ever in your life done any of these things or any type of work (not including housework)?*” Responses were coded as Yes (reference) and No.

Education was assessed by the total number of school years, categorized as no formal education (reference), primary schooling completed, secondary schooling completed, and college or more. In the SAGE study, income quintiles were estimated using assets and durable goods (*e.g.*, chairs, tables, and car; household ownership of electricity, television, phone, bucket, and washing machine), dwelling characteristics (type of floors, walls, and cooking stove), and services (*e.g.*, improved water, sanitation, and cooking fuel).^28^ Quintiles were created using the 21 assets and the lowest quintile was considered the reference category.

We examined social connectedness to understand the structure of social attachments (a mix of formal/informal, strong/weak ties and degrees of attachments) as well as the frame within which social support may be received. Respondents were asked about their involvement in the community in the social cohesion module. Participants were asked, how often in the last 12 months have you: “*had friends over to your home?,*” “*been in the home of someone who lives in a different neighborhood than you do or had them in your home?,*” “*socialized with coworkers outside of work?,*” “*attended religious services (not including weddings and funerals)?*” *and* “*gotten out of the house/your dwelling to attend social meetings, activities, programs, or events or to visit friends or relatives?*” Responses were reported as “never,” “once or twice per year,” “once or twice per month,” “once or twice per week,” and “daily.” We categorized these responses as “never,” “occasionally,” and “regularly.” We added these responses and created tertiles of social connectedness, which were coded as high (reference), moderate, and low.

Area of residence was categorized as rural (reference) and urban. India was the reference category for country in the analyses. The covariates of residence and country adjusted for crosscultural differences and unmeasured confounders that may influence caregiving as well as outcomes.

#### Analyses

We assessed sociodemographic characteristics of the WHO-SAGE sample for males and females across the five study countries and have reported prevalences of covariates by gender. We examined gender differences in the prevalence of measures of psychosocial wellbeing using two-sample *t*-tests (with level of significance). Our main study hypotheses included examining differences in the effects of caregiving on wellbeing among women and men, and if these were mediated by resources (socioeconomic factors) and social connectedness. For this, we examined gender and socioeconomic differences in informal caregiving with chi-square tests of association, as well as estimated gender differences and associations within categories of informal caregiving. We used multinomial regression models for the association between informal caregiving and feeling depressed, feeling anxiety, self-rated health, and self-rated experience of difficulties and used logistic regression for associations with diagnosed depression, adjusted for age, education, income, work status, current marital status, social connectedness, residence, and country in gender-stratified models. To examine robustness of self-reports, we compared diagnosed depression against affect measures of anxiety and depression. Regression analyses were weighted. All analyses were conducted using Stata version 13.0.^29^

## Results

Socioeconomic characteristics (as prevalences or column percentages) of males and females in the WHO-SAGE are reported in Tables 1 and 2, reporting associations of informal caregiving and the outcomes as well as covariates. Across countries, women were more likely to be informal caregivers (6.05%) compared with 4.97% of men (*p* < 0.0001) (Table 2). Differences in informal caregiving by age (younger respondents were more likely to be informal caregivers) (*p* = 0.007) and residence (rural residents were more likely to be caregivers) (*p* < 0.0001) were also noted. Participants who were currently married (6.47%) and participants who reported moderate social connectedness (6.61%) were more likely to be informal caregivers (*p* < 0.0001). Participants in India (7.6%) and Russia (7.88%) reported significantly higher rates of informal caregiving compared with Ghana (3.27%), Mexico (2.33%), and South Africa (2.47%) (*p* < 0.0001). Within countries, differences by gender and socioeconomic factors in caregiver characteristics were also noted with women more likely to be the main caregiver across all sampled countries with the exception of Ghana (Table 3).

**Table 1.** Characteristics of Adults (Total Numbers and Proportion by Sex) from Five Low- and Middle-Income Countries in the WHO-SAGE (2007–2010)

	Male,* n* = 11,734 (41.01%)	Female,* n* = 16,877 (58.99%)	Total,* n* = 28,611
Country			
India	4,677 (39.86)	7,441 (44.09)	12,118
Mexico	983 (8.38)	1,592 (9.43)	2,575
Russia	1,546 (13.18)	2,793 (16.55)	4,339
South Africa	1,742 (14.85)	2,339 (13.86)	4,081
Ghana	2,786 (23.74)	2,712 (16.07)	5,498
Age group (in years)			
18–49	1,986 (16.93)	5,059 (29.98)	7,045
50+	9,748 (83.07)	11,818 (70.02)	21,566
Highest level of education			
No formal schooling	3,001 (25.58)	6,612 (39.18)	9,613
Primary completed	3,309 (28.20)	4,214 (24.97)	7,523
Secondary completed	4,317 (36.79)	4,951 (29.34)	9,268
College or above	1,107 9.43)	1,100 (6.52)	2,207
Residence			
Rural	6,628 (56.49)	8,696 (51.53)	15,324
Urban	5,106 (43.51)	8,181 (48.47)	13,287
Marital status			
Never married	603 (5.14)	1,306 (7.74)	1,909
Currently married	9,707 (82.73)	9,336 (55.32)	19,043
Separated/widowed	1,424 (12.14)	6,235 (36.94)	7,659
Income quintile^a^			
Poorest (Q1)	2,033 (17.33)	3,233 (19.16)	5,266
Poorer (Q2)	2,232 (19.02)	3,330 (19.73)	5,562
Middle (Q3)	2,174 (18.53)	3,366 (19.94)	5,540
Richer (Q4)	2,491 (21.23)	3,438 (20.37)	5,929
Richest (Q5)	2,804 (23.90)	3,510 (20.80)	6,314
Ever worked			
Yes worked	10,621 (94.59)	10,839 (68.23)	21,460
Never worked	607 (5.41)	5,047 (31.77)	5,654
Social connectedness^b^			
High	3,095 (28.57)	3,089 (20.37)	6,184
Moderate	3,982 (36.75)	4,534 (29.89)	8,516
Low	3,757 (34.68)	7,545 (49.74)	11,302

China was excluded as data for caregiving were unavailable.

^a^ Income quintiles used as derived variable from the data, comprised 21 assets and amenities.

^b^ Details on composition of social connectedness are available in the Methods section.

WHO-SAGE, World Health Organization Study on Global AGEing, and Adult Health.

**Table 2.** Prevalence Differences in Informal Caregiving by Socioeconomic Characteristics (and Test of Association) for Adults in the WHO-SAGE Study (2007–2010)

	No long-term sick adult at home	1 (+) long-term sick adult at home		Chi square and p
		Respondent: not caregiver	Respondent: caregiver	
Male	10,771 (91.79)	380 (3.24)	583 (4.97)	73.7, <0.0001
Female	15,551 (92.14)	305 (1.81)	1,021 (6.05)	
Age group (in years)				
18–49	6,437 (91.37)	161 (2.29)	447 (6.34)	9.9, 0.007
50+	19,885 (92.21)	524 (2.43)	1,157 (5.36)	
Education				
No formal schooling	8,857 (92.14)	239 (2.49)	517 (5.38)	27.8, <0.0001
Primary completed	6,992 (92.94)	179 (2.38)	352 (4.68)	
Secondary completed	8,463 (91.31)	212 (2.29)	593 (6.40)	
College or above	2,010 (91.07)	55 (2.49)	142 (6.43)	
Residence				
Rural	13,938 (90.96)	476 (3.11)	910 (5.94)	80.2, <0.0001
Urban	12,384 (93.20)	209 (1.57)	694 (5.22)	
Marital status				
Never married	1,770 (92.72)	55 (2.88)	84 (4.40)	90.4, <0.0001
Currently married	17,335 (91.03)	476 (2.5)	1,232 (6.47)	
Separated/widowed	7,217 (94.23)	154 (2.01)	288 (3.76)	
Income quintile				
Poorest (Q1)	4,933 (93.68)	87 (1.65)	246 (4.67)	40.5, <0.0001
Poorer (Q2)	5,145 (92.50)	123 (2.21)	294 (5.29)	
Middle (Q3)	5,093 (91.93)	135 (2.44)	312 (5.63)	
Richer (Q4)	5,415 (91.33)	154 (2.60)	360 (6.07)	
Richest (Q5)	5,736 (90.85)	186 (2.95)	392 (6.21)	
Ever work				
Yes worked	19,635 (91.50)	545 (2.54)	1,280 (5.96)	0.5, 0.7
Never worked	5,190 (91.79)	140 (2.48)	324 (5.73)	
Social connectedness tertiles				
High	5,750 (92.98)	128 (2.07)	306 (4.95)	27.3, <0.0001
Med	7,715 (90.59)	238 (2.79)	563 (6.61)	
Low	10,367 (91.73)	291 (2.57)	644 (5.70)	
Country				
India	10,726 (88.51)	471 (3.89)	921 (7.60)	577.4, <0.0001
Mexico	2,493 (96.82)	22 (0.85)	60 (2.33)	
Russia	3,888 (89.61)	109 (2.51)	342 (7.88)	
South Africa	3,954 (96.89)	26 (0.64)	101 (2.47)	
Ghana	5,261 (95.69)	57 (1.04)	180 (3.27)	

**Table 3.** Prevalence Differences by Sex for Caregiving Status and Socioeconomic Determinants Along with Chi-Square Test of Association in the WHO-SAGE Study (2007–2010)

	No long-term sick adult at home		1 (+) long-term sick adult at home			
			Respondent is not the main caregiver		Respondent is the main caregiver	
	Male	Female	Male	Female	Male	Female
Country						
India	4,092 (38.04)	6,665 (61.96)	256 (54.47)	214 (45.53)	329 (36.92)	562 (63.08)
Mexico	953 (38.23)	1,540 (61.77)	15 (68.18)	7 (31.82)	15 (25.0)	45 (75.0)
Russia	1,373 (35.29)	2,518 (64.71)	67 (61.47)	42 (38.53)	106 (31.27)	233 (68.72)
South Africa	1,696 (42.87)	2,260 (57.13)	12 (46.15)	14 (53.85)	34 (34.34)	65 (65.66)
Ghana	2,674 (50.71)	2,599 (49.29)	30 (53.57)	26 (46.43)	82 (48.52)	87 (51.48)
Area of residence						
Rural	6,001 (42.95)	7,972 (57.05)	273 (57.59)	201 (42.41)	354 (40.36)	523 (59.64)
Urban	4,787 (38.61)	7,610 (61.39)	107 (51.20)	102 (48.80)	212 (31.13)	469 (68.87)
Age (in years)						
18–35	676 (22.56)	2,321 (77.44)	24 22.64)	82 (77.36)	42 (22.58)	144 (77.42)
35–59	4,808 (42.53)	6,498 (57.47)	166 (67.21)	81 (32.79)	261 (34.62)	493 (65.38)
60+	5,304 (43.95)	6,763 (56.05)	190 (57.58)	140 (42.42)	263 (42.56)	355 (57.44)
Education						
No formal school	2,759 (31.10)	6,112 (68.90)	102 (42.86)	136 (57.14)	140 (27.78)	364 (72.22)
Completed primary	3,064 (43.73)	3,942 (56.27)	106 (59.22)	73 (40.78)	139 (41.12)	199 (58.88)
Completed secondary	3,953 (46.61)	4,528 (53.39)	135 (63.98)	76 (36.02)	229 (39.76)	347 (60.24)
College or above	1,012 (50.30)	1,000 (49.70)	37 (67.27)	18 (32.73)	58 (41.43)	82 (58.57)
Marital status						
Never married	570 (32.15)	1,203 (67.85)	14 (25.45)	41 (74.55)	19 (23.46)	62 (76.54)
Currently married	8,899 (51.23)	8,481 (48.77)	314 (66.11)	161 (33.89)	494 (41.24)	704 (58.76)
Separated–widowed	1,319 (18.25)	5,908 (81.75)	52 (33.99)	101 (66.01)	53 (190.0)	226 (81.0)
Income quintile						
Poorest (Q1)	1,882 (38.12)	3,055 (61.88)	53 (60.92)	34 (39.08)	98 (40.50)	144 (59.50)
Poorer (Q2)	2,063 (40.03)	3,090 (59.97)	65 (52.85)	58 (47.15)	104 (36.36)	182 (63.64)
Middle (Q3)	2,004 (39.25)	3,102 (60.75)	67 (50.0)	67 (650.0)	103 (34.33)	197 (65.67)
Richer (Q4)	2,270 (41.84)	3,155 (58.16)	87 (56.86)	66 (43.14)	134 (38.18)	217 (61.82)
Richest (Q5)	2,569 (44.69)	3,180 (55.31)	108 (58.06)	78 (41.94)	127 (33.51)	252 (66.49)
Ever worked						
Yes worked	9,098 (49.30)	9,972 (50.70)	372 (68.51)	171 (31.49)	551 (44.19)	696 (55.81)
Never worked	589 (11.29)	4,627 (88.71)	8 (5.81)	132 (94.29)	15 (4.82)	296 (95.18)
Social network tertile						
High	4,366 (48.74)	4,592 (51.26)	138 (65.71)	72 (34.29)	214 (44.21)	270 (55.79)
Med	3,366 (40.59)	4,927 (59.41)	151 (56.34)	117 (43.66)	241 (38.19)	390 (61.81)
Low	2,173 (32.80)	4,452 (67.20	85 (48.02)	92 (51.98)	100 (28.09)	256 (71.91)

In the study sample, women were more likely to feel severely depressed (7.3% vs. 4.46% among men, *p* < 0.0001) and feel severe anxiety (10.64% vs. 7.29% for men, *p* < 0.0001); smaller gender differences were noted for diagnosed depression compared with self-reports (4.66% of women compared with 3.23% of men, *p* < 0.0001) (Table 4). Gender differences were also noted in self-rated health; 41.3% of men reported good self-rated health compared with 32.2% of women, while 15.16% of men reported poor self-rated health compared with 18.04% of women (*p* < 0.0001). Small but statistically significant differences by gender were also noted in self-reports of experienced difficulties (*p* < 0.0001). Validity and sensitivity analyses compared diagnosed depression with measures of affect yielding expected findings (Supplementary Table S1).

**Table 4.** Prevalence of Measures of Wellbeing for Adults by Sex and *t*-test of Association in the WHO-SAGE (2007–2010)

	Male	Female	p
Feeling depressed?			
None	6,269 (56.08)	7,841 (49.54)	<0.0001
Mild–mod	4,411 (39.46)	6,830 (43.15)	
Severe–extreme	498 (4.46)	1,156 (7.30)	
Feeling anxiety?			
None	5,577 (49.97)	6,702 (42.43)	<0.0001
Mild–mod	4,770 (42.74)	7,412 (46.93)	
Severe–extreme	814 (7.29)	1,680 (10.64)	
Self-rated health			
Good	4,633 (41.3)	5,431 (32.21)	<0.0001
Moderate	4,885 (43.54)	7,581 (47.75)	
Bad	1,701 (15.16)	2,864 (18.04)	
Self-rated awareness of difficulties^a^			
None	3,764 (33.60)	4,641 (29.29)	<0.0001
Mild	2,652 (23.67)	3,822 (24.11)	
Moderate	3,436 (30.67)	5,162 (32.57)	
Severe–extreme	1,350 (12.05)	2,225 (14.04)	
Diagnosed depression			
No	10,778 (96.77)	15,053 (95.34)	<0.0001
Yes	360 (3.23)	735 (4.66)	

^a^ Difficulties were defined as requiring increased effort, discomfort or pain, slowness, or changes in the way you do activities.

Multivariate models showing the associations of informal caregiving with outcomes, adjusted for sociodemographic and other covariates are presented in Tables 5–7. Compared with participants who reported no one ill at home, female caregivers reported higher severe–extreme depression (adjusted relative risk ratios [ARRR] = **1.86** confidence interval [95% CI: 1.28–2.69]) while estimates for feeling depressed among men were not statistically significant (Table 3). Reports of anxiety were greater among male caregivers who reported higher mild–moderate anxiety (ARRR = **1.8** [95% CI: 1.2–2.69]) and higher likelihood of severe–extreme anxiety (ARRR = **2.22** [95% CI: 1.07–4.58]), whereas female caregivers reported higher mild–moderate anxiety (ARRR = **1.64** (95% CI: 1.22–2.22]) compared with female noncaregivers (Table 5). Informal caregiving was not associated with poor self-rated health for men or women (Table 6). Female caregivers reported higher moderate difficulties with life tasks (ARRR = 1.45 [95% CI: 1.01–2.08]) compared with female noncaregivers, but the same was not seen for male caregivers. However, having someone sick at home was associated with higher reporting of extreme difficulties in life tasks for women (ARRR = **2.32** [1.33–4.04]) (Table 6). Caregiving status was not statistically significant with diagnosed depression among male caregivers in adjusted models (adjusted odds ratio [AOR] = 1.25 [0.6–2.5]) (Table 7).

**Table 5.** Regression Results from Multinomial Regression Models Presenting Relative Risk Ratios and 95% Confidence Intervals for Feeling Depressed and Feeling Anxiety by Sex in the WHO-SAGE Study (2007–2010)

	Feel depressed				Feel anxiety			
	Male		Female		Male		Female	
	Mild*–*moderate	Severe*–*extreme	Mild*–*moderate	Severe*–*extreme	Mild*–*moderate	Severe*–*extreme	Mild*–*moderate	Severe*–*extreme
Caregiving status: no illness	1.00	1.00	1.00	1.00	1.00	1.00	1.00	1.00
Respondent not main caregiver	1.05 (0.6–1.8)	1.02 (0.47–2.19)	1.08 (0.47–2.49)	2.08 (0.97–4.45)	0.86 (0.51–1.45)	0.88 (0.38–2.009)	1.01 (0.46–2.19)	1.21 (0.57–2.57)
Respondent main caregiver	1.40 (0.96–2.04)	2.29 (0.75–6.96)	1.18 (0.84–1.66)	1.86^*^(1.28–2.69)	1.80^*^(1.20–2.69)	2.22^*^(1.07–4.58)	1.64^*^(1.22–2.22)	1.44 (0.99–2.07)
Education								
No formal schooling	1.00	1.00	1.00	1.00	1.00	1.00	1.00	1.00
Primary completed	1.03 (0.74–1.43)	0.60 (0.32–1.11)	0.96 (0.8–1.17)	0.90 (0.63–1.28)	0.92 (0.66–1.29)	0.83 (0.50–1.38)	0.84 (0.68–1.04)	0.76 (0.56–1.03)
Secondary completed	0.7^*^(0.50–0.97)	0.74 (0.35–1.56)	0.78 (0.61–1.01)	0.61^*^(0.38–0.98)	0.82 (0.59–1.14)	0.74 (0.43–1.25)	0.70^*^(0.55–0.90)	0.62^*^(0.42–0.91)
College or above	0.78 (0.5–1.23)	0.41 (0.13–1.32)	0.62^*^(0.43–0.90)	0.17^*^(0.07–0.41)	0.95 (0.60–1.51)	0.71 (0.34–1.49)	0.49^*^(0.33–0.74)	0.13^*^(0.06–0.26)
Ever worked								
Yes	1.00	1.00	1.00	1.00	1.00	1.00	1.00	1.00
No	0.75 (0.41–1.64)	1.24 (0.40–3.84)	0.76^*^(0.64–0.91)	0.87 (0.62–1.23)	0.86 (0.46–1.62)	1.31 (0.56–3.05)	0.80^*^(0.66–0.96)	0.94 (0.71–1.25)
Age (in years)								
18–49	1.00	1.00	1.00	1.00	1.00	1.00	1.00	1.00
50+	1.99^*^(1.59–2.5)	3.26^*^(2.0–5.29)	1.48^*^(1.19–1.85)	1.47^*^(1.09–1.99)	1.71^*^(1.35–2.18)	3.08^*^(2.19–4.34)	1.43^*^(1.10–1.84)	1.49^*^(1.13–1.96)
Residence								
Rural	1.00	1.00	1.00	1.00	1.00	1.00	1.00	1.00
Urban	0.69^*^(0.53–0.91)	0.95 (0.5–1.81)	0.79 (0.61–1.04)	0.87 (0.56–1.37)	0.69^*^(0.52–0.91)	0.75 (0.41–1.36)	0.90 (0.69–1.17))	1.10 (0.70–1.72)
Marital status								
Never married	1.00	1.00	1.00	1.00	1.00	1.00	1.00	1.00
Currently married	1.04 (0.66–1.64)	1.31 (0.40–4.25)	1.68^*^(1.18–2.39)	3.0^*^(1.41–6.35)	1.61^*^(1.06–2.42)	1.55 (0.70–3.44)	1.75^*^(1.27–2.42)	4.49^*^(2.24–9.0)
Separated–widowed	1.68 (0.88–3.19)	3.13 (0.81–11.9)	2.95^*^(1.99–4.37)	10.9^*^(4.9–24.5)	2.14^*^(1.14–3.99)	3.49^*^(1.12–10.8)	2.55^*^(1.72–3.76)	12.6^*^(5.7–27.9)
Social connectedness: high	1.00	1.00	1.00	1.00	1.00	1.00	1.00	1.00
Med	0.93 (0.70–1.22)	0.75 (0.38–1.47)	0.70^*^(0.55–0.90)	0.53^*^(0.30–0.93)	0.83 (0.61–1.14)	0.97 (0.58–1.63)	0.78 (0.59–1.03)	0.60 (0.38–0.94)
Low	1.41^*^(1.02–1.96)	2.44^*^(1.24–4.79)	0.89 (0.66–1.14)	0.79 (0.45–1.50)	1.20 (0.85–1.69)	2.63^*^(1.49–4.63)	1.009 (0.77–1.31)	0.89 (0.57–1.38)

Estimates were weighted. RRRs adjusted for income and country. Reference category is None.

^*^ and values in bold indicates *p* < 0.05.

RRRs, relative risk ratios.

**Table 6.** Results from Multinomial Regression Models Presenting and 95% Confidence Intervals for Self-Rated Health and Experience of Difficulties by Sex in the WHO-SAGE Study (2007–2010)

	Self-rated health		Experience of difficulties			
	Male	Female	Male		Female	
	Bad/poor	Bad/poor	Moderate	Extreme	Moderate	Extreme
Caregiving status: no illness	1.00	1.00	1.00	1.00	1.00	1.00
Respondent not main caregiver	1.57 (0.74–3.35)	1.44 (0.81–2.57)	1.70 (0.75–3.85)	1.56 (0.64–3.79)	1.38 (0.75–2.53)	2.32^*^(1.33–4.04)
Respondent main caregiver	1.37 (0.69–2.74)	1.09 (0.77–1.55)	0.97 (0.60–1.57)	0.64 (0.35–1.17)	1.45^*^(1.01–2.08)	1.27 (0.77–2.07)
Education						
No formal schooling	1.00	1.00	1.00	1.00	1.00	1.00
Primary completed	1.26 (0.80–1.98)	1.32^*^ (1.06–1.65)	1.07 (0.75–1.52)	1.13 (0.71–1.81)	0.84 (0.64–1.09)	0.91 (0.66–1.25)
Secondary completed	0.81 (0.52–1.27)	0.83 (0.56–1.21)	0.71 (0.48–1.05)	0.68 (0.41–1.13)	0.42^*^(0.31–0.57)	0.47^*^(0.30–0.73)
College or above	0.36^*^(0.17–0.76)	0.20^*^(0.12–0.35)	0.45^*^(0.25–0.8)	0.37^*^(0.18–0.77)	0.19^*^(0.11–0.35)	0.13^*^(0.05–0.31)
Ever worked						
Yes	1.00	1.00	1.00	1.00	1.00	1.00
No	1.56 (0.76–3.21)	1.39^*^(1.10–1.76)	1.09 (0.53–2.23)	1.52 (0.72–3.2)	0.66^*^(0.52–0.85)	1.03 (0.76–1.39)
Age (in years)						
18–49	1.00	1.00	1.00	1.00	1.00	1.00
50+	4.88^*^(3.41–7.0)	2.06^*^(1.61–2.65)	4.33^*^(3.23–5.79)	8.69^*^(5.97–12.6)	4.46^*^(3.36–5.93)	7.19^*^(4.98–10.3)
Residence						
Rural	1.00	1.00	1.00	1.00	1.00	1.00
Urban	0.98 (0.62–1.56)	0.88 (0.68–1.15)	0.69^*^(0.48–0.99)	0.91 (0.58–1.41)	0.97 (0.70–1.32)	0.70 (0.46–1.05)
Marital status						
Never married	1.00	1.00	1.00	1.00	1.00	1.00
Currently married	2.06 (0.99–4.28)	1.44 (0.77–2.67)	1.08 (0.64–1.81)	1.38 (0.62–3.09)	1.85^*^(1.16–2.95)	3.17^*^(1.48–6.78)
Separated–widowed	4.30^*^(1.79–10.2)	2.1^*^(1.12–3.95)	1.47 (0.70–3.08)	2.8^*^(1.06–7.39)	2.35^*^(1.42–3.9)	7.03^*^(3.19–15.4)
Social connectedness: high	1.00	1.00	1.00	1.00	1.00	1.00
Med	1.16 (0.73–1.85)	0.56^*^(0.38–0.82)	1.06 (0.72–1.57)	0.55^*^(0.32–0.94)	0.83 (0.60–1.16)	0.55^*^(0.36–0.84)
Low	2.67^*^(1.66–4.29)	0.87 (0.59–1.27)	1.34 (0.87–2.08)	1.47 (0.86–2.49)	0.85 (0.62–1.17)	0.76 (0.52–1.12)

Estimates were weighted. RRRs adjusted for income and country.

^*^ and values in bold indicates *p* < 0.05.

**Table 7.** Results from Logistic Regression Models (Odds Ratio and 95% Confidence Intervals) for Diagnosed Repression by Sex in the WHO-SAGE (2007–2010)

	Male	Female
Caregiving status: no illness	1.00	1.00
Respondent not main caregiver	0.46 (0.13–1.53)	0.08 (0.01–0.34)
Respondent main caregiver	1.25 (0.6–2.57)	0.70 (0.32–1.53)
Education		
No formal schooling	1.00	1.00
Primary completed	0.73 (0.34–1.57)	1.29 (0.82–2.04)
Secondary completed	0.69 (0.29–1.61)	1.80 (0.94–3.45)
College or above	0.70 (0.21–2.31)	1.63 (0.98–2.72)
Ever worked		
Yes	1.00	1.00
No	1.28 (0.62–2.65)	0.86 (0.54–1.37)
Age (in years)		
18–49	1.00	1.00
50+	0.90 (0.61–1.32)	1.44 (0.73–2.85)
Residence		
Rural	1.00	1.00
Urban	0.82 (0.41–1.62)	1/62 (0.98–2.72)
Marital status		
Never married	1.00	1.00
Currently married	2.14 (0.81–5.65)	4.74^*^(1.40–15.9)
Separated–widowed	2.03 (0.56–7.35)	6.88^*^(1.80–26.19)
Social connectedness: high	1.00	1.00
Med	1.22 (0.66–2.25)	1.71 (0.89–3.29)
Low	1.42 (0.73–2.76)	2.07^*^(1.07–4.02)

Estimates were weighted. Adjusted odds ratios adjusted for income and country. Reference in No.

^*^ and values in bold indicates *p* < 0.05.

Education was protective against feeling depressed or feeling anxiety, particularly among college-educated women (for mild–moderate depression, ARRR = **0.62** [95% CI: 0.4–0.9], for severe–extreme depression: ARRR = **0.18** [95% CI: 0.07–0.4]; for mild–moderate anxiety: ARRR = **0.49** [95% CI: 0.3–0.7]; for severe–extreme anxiety: ARRR = **0.13** [95% CI: 0.06–0.2]) (Table 5). Education was also protective against poor self-rated health and difficulties experienced by both men and women (Table 6).

Never worked status was associated with higher depression and anxiety among men; however estimates were not statistically significant. However, among women, never having worked was protective against feeling mild–moderate depressed (ARRR = **0.76** [0.6–0.9]) and feeling mild–moderate anxiety (ARRR = **0.8** [0.66–0.9]) (Table 5). Lower social connectedness was associated with feeling higher severe–extreme depressed among men (ARRR = **2.44** [1.24–4.79]) and among women (ARRR = **2.63** [1.49–4.63]) (Table 5). Lower social connectedness was also associated with poorer self-rated health among males (ARRR = 2.67 [1.66–4.29]) (Table 6) and higher diagnosed depression among females (OR = 2.07 [1.07–4.02]) (Table 7).

## Discussion

Our study showed that across LMICs, long-term informal caregiving was associated with poorer health and wellbeing, and women were not only more likely than men to be informal caregivers but that caregiving effects were more pronounced for female relative to male caregivers. Female caregivers were 1.86 times more likely to report severe depression and 1.45 times more likely to experience moderate difficulties in life tasks compared with female noncaregivers. These effects were not seen for men. Greater anxiety was reported by both male and female caregivers. Findings on the caregiver burden on depression and self-rated difficulties are consistent with previous evidence that shows high psychosocial and emotional costs of long-term caring on the wellbeing of caregivers.^14,30^ However, in addition, results contrasting depression with anxiety show some evidence that the nature, pathways, and outcomes of stress may vary across men and women. Findings from this study are in line with previous accounts that show higher health burdens of caregiving for women; we additionally show that caregiver distress may be experienced differently by men and women. Differences in the stress process for women and men may be attributed to gender socialization,^30–33^ social patterns of reinforcement, and purposeful coping behaviors when faced with situations,^12,14,19,30^ gender differences in perception and reporting of care activities,^12,19^ undertaken (*e.g.*, cooking, bathing, and dressing, although differences are small^19,24,34^), and valued. In LMICs, evidence on the gendered nature of these stress pathways is lacking and further research is needed through time use and mixed methods studies.

Informal caregivers, presently unrecognized, are synergistic to formal health systems^35–37^ and services and allowances need to be created for them as extensions to the health system.^38,39^ Present economic estimates of the contributions of informal caregivers, particularly women comes from HICs and needs systematic study in LMICs. Caregivers face a range of challenges, including the absence of institutional support and limited financial protection and care responsibilities fall disproportionately on women^18,20,34,40^ and losses in livelihood and dropping out of the workforce.^8^ Female caregivers often experience social pressures such as the obligation to value the needs of a family member over one's own.^19,32^ Gender differences in social support are also documented^41–43^ showing males as better socially connected and able to leverage support and help easier than females, thus able to better cope with pressures of care.^30^

In this study, we found that low social connectedness influenced affect and self-rated health among males, and diagnosed depression among females. Social connectedness, a measure of social resources, may be differently valued by men and women, or offer different opportunities and advantages to them. Higher education was also found to be protective against negative affect measures, poor self-rated health, and experience of difficulties for both men and women, highlighting its potential role in buffering stress. In-depth accounts of social support received through the networks can be useful in more insightful understanding of how stress is mediated for men and women.

This study uses data from the WHO-SAGE, one of the few crossnational studies to assess informal caregiving and wellbeing. SAGE uses established standards and protocols, and collects nationally representative data, thereby providing inferences with high validity and reliability. Study data were designed and implemented by the WHO-SAGE team and authors had access to public use data. Findings need to be considered in light of the following considerations. First, study authors did not have detailed information on the care recipient and illness status, and therefore inferences were limited by availability of data. This also limited the constructs from the theoretical framework that could be tested. We recommend that future studies provide more detail on the care recipient to understand the dynamics and strains of care. Second, we did not have data to investigate within-country culturally specific stress process trajectories in the sample. Cultural context can play a role in the meaning and reporting of mental health outcomes,^44^ in setting caregiving expectations as well as in determining fulfillment associated with fulfilling role obligations.^7,31^ Data provided an opportunity to understand the issue crossnationally and we recommend future studies on aging and caregiving, to study the cultural and social elements of this issue within national contexts. Investigating these country-specific trajectories can provide deeper insight into social processes that can influence caregiver burden.^45–47^ For instance, the Longitudinal Study on Aging in India (LASI) is an effort to study aging in India in the coming years; similar efforts are needed across other countries. Third, data are based on self-reports which may be prone to recall bias. This bias may be somewhat reduced by shorter recall periods like 30 days that were used in this study but improving measurement of caregiving and distress outcomes is a concern and needs greater push. Fourth, in the study, we used social connectedness to understand social resources available to respondents.^3^ We recognize though that connectedness may not always lead to social support. Finally, WHO-SAGE data are cross-sectional and our analyses infer associations but do not imply causality. Collection of longitudinal data by LMICs will facilitate a richer understanding of the stress process and lead to development of distress reduction interventions.

## Conclusion

Long-term informal caregiving is associated with worse health outcomes, particularly greater mental health burdens in this sample of older adults from Ghana, India, Mexico, South Africa, and Russia. These burdens disproportionately affect women more than men, even as our findings reveal that distress is experienced by men on some outcomes more than others. Female caregivers are more likely to report greater depression from informal caregiving, and both male and female caregivers report higher anxiety compared with noncaregivers. More research is needed to understand cultural factors and gender norms that determine ways in which responsibilities and expectations of providing informal care are experienced, internalized, and expressed. Interventions to support caregivers, particularly women who provide unpaid care work, are urgently needed in LMICs that are likely to face a growing burden of care with rising burdens of non-communicable diseases and growing older populations.

## Supplementary Material

Supplemental data

## References

[1] Haines A, Sanders D, Lehmann U, et al. Achieving child survival goals: Potential contribution of community health workers. Lancet 2007;369:2121–2131 17586307 10.1016/S0140-6736(07)60325-0

[2] Pallas SW, Minhas D, Pérez-Escamilla R, Taylor L, Curry L, Bradley EH Community health workers in low-and middle-income countries: What do we know about scaling up and sustainability? Am J Public Health 2013;103:e74–e82 10.2105/AJPH.2012.301102 PMC3682607 23678926

[3] Pearlin LI, Mullan JT, Semple SJ, Skaff MM Caregiving and the stress process: An overview of concepts and their measures. Gerontologist 1990;30:583–594 2276631 10.1093/geront/30.5.583

[4] Bhattacharjee M, Vairale J, Gawali K, Dalal PM Factors affecting burden on caregivers of stroke survivors: Population-based study in Mumbai (India). Ann Indian Acad Neurol 2012;15:113 22566724 10.4103/0972-2327.94994 PMC3345587

[5] Sanyal J, Das S, Ghosh E, Banerjee T, Bhaskar L, Rao VR Burden among Parkinson's disease care givers for a community based study from India. J Neurol Sci 2015;358:276–281 26382831 10.1016/j.jns.2015.09.009

[6] Nortey ST, Aryeetey GC, Aikins M, Amendah D, Nonvignon J Economic burden of family caregiving for elderly population in southern Ghana: The case of a peri-urban district. Int J Equity Health 2017;16:16 28088236 10.1186/s12939-016-0511-9 PMC5237474

[7] Scharlach AE, Kellam R, Ong N, Baskin A, Goldstein C, Fox PJ Cultural attitudes and caregiver service use: Lessons from focus groups with racially and ethnically diverse family caregivers. J Gerontol Soc Work 2006;47:133–156 16901881 10.1300/J083v47n01_09

[8] Sharma N, Chakrabarti S, Grover S Gender differences in caregiving among family-caregivers of people with mental illnesses. World J Psychiatry 2016;6:7 27014594 10.5498/wjp.v6.i1.7 PMC4804270

[9] Morris M. Gender-sensitive home and community care and caregiving research: A synthesis paper. Centers of Excellence for Women's Health 2001. Available at: www.womenandhealthcarereform.ca/publications/synthesis.pdf Accessed 121, 2018

[10] Mathiowetz NA, Oliker S The gender gap in caregiving to adults. Manuscript prepared for presentation at the American Time Use Survey Early Results Conference. University of Wisconsin-Milwaukee 2005–09. Available at: www.atususers.umd.edu/wip2/papers/Oliker.pdf Accessed 121, 2018

[11] Reinhard SC, Feinberg LF, Choula R, Houser A Valuing the invaluable: 2015 update. Insight on the Issues. 2015;104:89–98

[12] Etters L, Goodall D, Harrison BE Caregiver burden among dementia patient caregivers: A review of the literature. J Am Acad Nurse Pract 2008;20:423–428 18786017 10.1111/j.1745-7599.2008.00342.x

[13] Bevans M, Sternberg EM Caregiving burden, stress, and health effects among family caregivers of adult cancer patients. JAMA 2012;307:398–403 22274687 10.1001/jama.2012.29 PMC3304539

[14] Smale B, Dupuis SE Caregivers of persons with dementia: Roles, experiences, supports and coping. In: Murray Alzheimer Research and Education Program, University of Waterloo, MAREP 2004

[15] Grunfeld E, Coyle D, Whelan T, et al. Family caregiver burden: Results of a longitudinal study of breast cancer patients and their principal caregivers. CMAJ 2004;170:1795–1801 15184333 10.1503/cmaj.1031205 PMC419766

[16] Kwok T, Bel Wong II, Chui K, Young D, Ho F Telephone-delivered psychoeducational intervention for Hong Kong Chinese dementia caregivers: A single-blinded randomized controlled trial. Clin Interv Aging 2013;8:1191 24072965 10.2147/CIA.S48264 PMC3783504

[17] Yee JL, Schulz R Gender differences in psychiatric morbidity among family caregivers: A review and analysis. Gerontologist 2000;40:147–164 10820918 10.1093/geront/40.2.147

[18] Parks SH, Pilisuk M Caregiver burden: Gender and the psychological costs of caregiving. Am J Orthopsychiatry 1991;61:501 1746626 10.1037/h0079290

[19] Kramer BJ, Kipnis S Eldercare and work-role conflict: Toward an understanding of gender differences in caregiver burden. Gerontologist 1995;35:340–348 7622087 10.1093/geront/35.3.340

[20] Gallicchio L, Siddiqi N, Langenberg P, Baumgarten M Gender differences in burden and depression among informal caregivers of demented elders in the community. Int J Geriatr Psychiatry 2002;17:154–163 11813279 10.1002/gps.538

[21] Jacobs JVH, Van Houtven CH, Laporte A, Coyte PC The impact of informal caregiving intensity on women's retirement in the United States, Working Paper 2014-09. Toronto, Canada: Canadian Center for Health Economics, 2014

[22] Välimäki TH, Vehviläinen-Julkunen KM, Pietilä A-MK, Pirttilä TA Caregiver depression is associated with a low sense of coherence and health-related quality of life. Aging Mental Health 2009;13:799–807 19888700 10.1080/13607860903046487

[23] Hansen T, Slagsvold B Feeling the squeeze? The effects of combining work and informal caregiving on psychological well-being. Eur J Ageing 2015;12:51–60 28804345 10.1007/s10433-014-0315-y PMC5549215

[24] Knight BG, Sayegh P Cultural values and caregiving: The updated sociocultural stress and coping model. J Gerontol B 2010;65:5–13 10.1093/geronb/gbp096 19934166

[25] Kowal P, Chatterji S, Naidoo N, et al. Data resource profile: The World Health Organization Study on global AGEing and adult health (SAGE). Int J Epidemiol 2012;41:1639–1649 23283715 10.1093/ije/dys210 PMC3535754

[26] WHO. WHO Study on global AGEing and adult health (SAGE). 2011. Available at: https://www.who.int/healthinfo/sage/en/ Accessed 61, 2018

[27] WHO. WHO Multi-Country Studies Data Archive. Available at: http://appswhoint/healthinfo/systems/surveydata/indexphp/catalog

[28] Arokiasamy P, Uttamacharya U, Jain K, et al. The impact of multimorbidity on adult physical and mental health in low-and middle-income countries: What does the study on global ageing and adult health (SAGE) reveal? BMC Med 2015;13:178 26239481 10.1186/s12916-015-0402-8 PMC4524360

[29] StataCorp. Stata Statistical Software: Release 13. 2013

[30] McLean CP, Anderson ER Brave men and timid women? A review of the gender differences in fear and anxiety. Clin Psychol Rev 2009;29:496–505 19541399 10.1016/j.cpr.2009.05.003

[31] Chakrabarti S, Penadés R, Catalán R Cultural aspects of caregiver burden in psychiatric disorders. World J Psychiatry 2013;3:85–92

[32] Miller B. Gender differences in spouse caregiver strain: Socialization and role explanations. J Marriage Fam 1990:311–321

[33] Miller B, Cafasso L Gender differences in caregiving: Fact or artifact? Gerontologist 1992;32:498–507 1427253 10.1093/geront/32.4.498

[34] Barusch AS, Spaid WM Gender differences in caregiving: Why do wives report greater burden? Gerontologist 1989;29:667–676 2513266 10.1093/geront/29.5.667

[35] Thrush A, Hyder A The neglected burden of caregiving in low-and middle-income countries. Disabil Health J 2014;7:262–272 24947567 10.1016/j.dhjo.2014.01.003

[36] Martin CD. More than the work: Race and gender differences in caregiving burden. J Fam Issues 2000;21:986–1005

[37] Levitsky SR. Caring for our own: Why there is no political demand for new American social welfare rights. USA: Oxford University Press, 2014

[38] Omaswa F. Informal health workers: To be encouraged or condemned? Bull World Health Organ 2006;84:83 16501720 10.2471/blt.05.027979 PMC2626536

[39] Donelan K, Hill CA, Hoffman C, et al. Challenged to care: Informal caregivers in a changing health system. Health Aff 2002;21:222–231 10.1377/hlthaff.21.4.222 12117133

[40] Langer A, Meleis A, Knaul FM, et al. Women and health: The key for sustainable development. Lancet 2015;386:1165–1210 26051370 10.1016/S0140-6736(15)60497-4

[41] Shumaker SA, Hill DR Gender differences in social support and physical health. Health Psychol 1991;10:102 2055208 10.1037//0278-6133.10.2.102

[42] Neff LA, Karney BR Gender differences in social support: A question of skill or responsiveness? J Pers Soc Psychol 2005;88:79 15631576 10.1037/0022-3514.88.1.79

[43] Reevy GM, Maslach C Use of social support: Gender and personality differences. Sex Roles 2001;44:437–459

[44] Groleau D, Pluye P, Nadeau L A mix-method approach to the cultural understanding of distress and the non-use of mental health services. J Mental Health 2007;16:731–741

[45] Ugargol AP, Bailey A Family caregiving for older adults: Gendered roles and caregiver burden in emigrant households of Kerala, India. Asian Popul Stud 2018;14:194–210

[46] Yakubu, Y.A., Schutte, D.W. Caregiver attributes and socio-demographic determinants of caregiving burden in selected low-income communities in cape town, South Africa. J of Compassionate Health Care 2018;5,3. 10.1186/s40639-018-0046-6.

[47] Trapp SK, Ertl MM, Gonzalez-Arredondo S, Rodriguez-Agudelo Y, Arango-Lasprilla JC Family cohesion, burden, and health-related quality of life among Parkinson's disease caregivers in Mexico. Int Psychogeriatr 2019;31:1039–1045 10.1017/S1041610218001515 30318024

