# Supplementary material for: Gender Differences in the Associations Between Informal Caregiving and Wellbeing in Low- and Middle-Income Countries
Source: J Womens Health (Larchmt). 2020 Oct 14;29(10):1328–38. doi: 10.1089/jwh.2019.7769 (PMC7583325; doi:10.1089/jwh.2019.7769)
Supplement: Supplemental data [file Suppl_TableS1.pdf]

Supplementary Data

Supplementary Table S1. Comparing Affect Measures (Feeling Depressed and Feeling Anxiety) Against Diagnosed Depression Among Adults in the WHO-SAGE Study (2007–2010)

	Diagnosed depression			
	No	*Yes*	*Total*	*Chi-square and* p
Feeling sad or depressed				
None	13,751 (98.26)	243 (1.74)	13,994	637.1, <0.0001
Mild–moderate	10,554 (94.38)	628 (5.62)	11,182	
Severe–extreme	1,423 (86.50)	222 (13.50)	1,645	
Feeling worried or anxiety				
None	11,982 (98.41)	194 (1.59)	12,176	628.3, <0.0001
Mild–moderate	11,515 (95.09)	595 (4.91)	12,110	
Severe–extreme	2,183 (87.85)	302 (12.15)	2,485	

WHO-SAGE, World Health Organization Study on Global AGEing and Adult Health.
